# Research on Integrated Technologies for Space Target Imaging, Ranging, and Communication

**DOI:** 10.3390/jimaging12070292

**Published:** 2026-06-30

**Authors:** Xiansong Gu, Qiang Fu, Zhuang Liu, Guan Wang, Hairui Wang, Chao Wang, Tianshu Wang, Yingchao Li, Huilin Jiang

**Affiliations:** 1 National and Local Joint Engineering Research Center of Space Optoelectronics Technology, Changchun 130022, China; emperorgxs@sina.com (X.G.); zhuangzhilingyun2007@aliyun.com (Z.L.); nicklo19992009@163.com (C.W.); wangts@cust.edu.cn (T.W.); 13944068295@163.com (Y.L.); hljiang@cust.edu.cn (H.J.); 2 School of Optoelectronic Engineering, Changchun University of Science and Technology, Changchun 130022, China; 13942130320@163.com (G.W.); hurrywang0612@163.com (H.W.)

**Keywords:** laser ranging, laser imaging, laser communication, Cassegrain optical system

## Abstract

The integration requirements of laser ranging, imaging, and communication functions in space target detection have placed higher demands on system performance. This paper takes a modularly designed integrated laser ranging, imaging, and communication system as an example and proposes a light source integration scheme based on fiber phased array beam splitting–coupling technology, effectively enhancing the system’s integration level and compactness. The system employs a Cassegrain optical system and beam splitting structure to achieve functional integration of laser communication, ranging, and polarization imaging. Ground experiments were conducted to evaluate the functional feasibility of the proposed integrated architecture. The visible light polarization imaging experiments at kilometer-level distances demonstrate that polarization-derived information can improve target–background separability under haze and low-contrast conditions. The UAV-based dynamic ranging experiment verifies that the system can acquire, track, and range a moving cooperative target under the tested field conditions, with the measured results being consistent with the designed meter-level ranging requirement. In addition, a 1 km coherent free-space laser communication experiment achieved 20 Gbps QPSK signal transmission with a bit error rate on the order of 10^−7^. These results provide experimental support and design references for integrated optoelectronic terminals used in space target observation, space debris monitoring, and related long-distance sensing and communication applications.

## 1. Introduction

With the continuous development and advancement of information transmission technology using laser carriers, laser carrier technology has been widely applied in spatial information transmission missions. Equipping platforms such as satellites or aircraft with laser carrier technologies, including laser communication, ranging, and imaging systems, enables the provision of wide-range observation, high-precision measurement, and communication capabilities. Laser communication refers to a technology that utilizes lasers as carriers for transmitting information such as voice, images, and data. After years of exploration, spatial laser communication has achieved breakthrough progress in recent years. Compared with radio frequency (RF) communication, laser communication operates at a frequency four to five orders of magnitude higher, which endows it with distinct advantages, including large communication capacity, small system volume, wide bandwidth, highly concentrated energy, and relatively low transmission power. As an effective means to address the high-speed bottleneck of microwave communication, laser communication exhibits enormous potential in both military and civilian applications. It is regarded as the optimal approach for high-speed data transmission between various platforms, such as satellite-to-satellite, ground-to-satellite, and aircraft-to-ground links. Laser ranging technology, which accurately measures the distance to a target using lasers, is one of the earliest and most widely adopted laser technologies in military fields. Classified by its ranging method, it can be divided into two categories: pulsed laser ranging and continuous wave laser ranging. With the steady development of laser communication and laser ranging technologies, the design and implementation of an integrated system incorporating laser communication, laser ranging, and imaging functions can enable multiple platforms (e.g., satellites, aircraft, and ground stations) to achieve diverse capabilities, including laser communication, ranging, and imaging.

The integrated laser communication, ranging, and imaging system is mainly divided into three parts: laser communication, laser ranging, and imaging. Laser communication is characterized by large information capacity, wide bandwidth, strong anti-electromagnetic interference ability, and good confidentiality; laser ranging has the characteristics of high power, good stability, and high precision; tracking and imaging technology has the advantage of stable tracking and imaging of targets. Combining the functions of the three to form an integrated system device, the integrated system can be mounted on various platforms such as aircraft, satellites, vehicles, and ships. Therefore, the integrated laser communication, ranging, and imaging system has broad application prospects and great practical value.

Due to the certain requirements of space exploration systems on their own weight and load capacity, the system must achieve high integration and multi-functionalization. Considerable efforts have been made at home and abroad to this end. A typical example is the American X2000 flight terminal, which can realize functions such as two-way communication, two-way laser ranging, and scientific imaging [1]. In the Integrated Laser Communication and Space Imaging (ACLAIM) program of the Jet Propulsion Laboratory, the laser communication antenna and the space camera share a front-end telescope, using a detector array as the acquisition and tracking system and simultaneously as the imaging receiver. In 2005, John J. Degnan proposed a renovation plan for the SLR2000 satellite laser ranging station, which combines laser ranging and laser communication. Specifically, the ranging light of the SLR2000 laser rangefinder is used as the beacon light for laser communication to realize tracking and aiming, which fully reflects the idea of integration of laser measurement and communication [2]. In 2010, the 504th Institute of Aerospace Science and Technology adopted asynchronous transponder laser ranging and communication technology [3]. In 2011, Jiang Huilin et al. proposed a multi-functional optical system that can simultaneously realize laser ranging, multi-dimensional imaging, and laser communication. On this basis, an integrated ranging, imaging, and communication system for detecting space debris was designed, which uses a catadioptric hybrid Cassegrain system to achieve ranging, imaging, and communication [4]. In 2015, Germany established a vehicle-mounted adaptive optical communication ground station, which realized high-rate transmission between the vehicle-mounted adaptive laser communication terminal and LEO with a transmission rate of 5.625 Gb/s. At the same time, it achieved two-way laser communication with a bandwidth of 2.8125 Gb/s and an effective rate of 1.8 Gb/s between the laser communication terminal of the geosynchronous satellite Alpha sat [5]. In March 2021, ASTROSCALE planned to launch its first mission to actively remove orbital debris, with the payload named “Servicer” [6]. In 2025, experiments on short-range atmospheric laser communication conducted by Changchun University of Science and Technology showed that Airy-like hypergeometric Gaussian beams perform the best in weak and moderate turbulence environments, with less influence from turbulence than other beams, and Bessel–Gaussian beams have better effects in strong turbulence [7]. In the same year, the Jiangsu International Joint Laboratory of Gallium Nitride Optoelectronic Integration proposed a laser communication system, which is conducive to promoting low-cost, high-speed, and long-distance visible light communication systems [8]. Also in 2025, the original 1 m laser communication telescope system at the Changchun Station of the Artificial Satellite Observatory was renovated to add the ranging capability for space debris targets. The measured data showed that the maximum accuracy of effective data is better than 1 m. For the optical design of space debris detection, the catadioptric optical system with shared primary and secondary mirrors for multi-spectral detection (visible, MWIR, LWIR) has been verified to have excellent imaging performance in the space temperature environment, which provides an important optical structural reference for the design of space-based debris detection payloads [9]. In 2026, Changchun University of Science and Technology proposed a Doppler frequency shift link simulation scheme based on the electro-optic effect of lithium niobate crystal and a dual-polarization parallel structure IQ modulator [10].

Despite these advances, most existing integrated optical terminals mainly realize functional coexistence by sharing a telescope aperture or by combining several independent modules at the system level [1,2,3,4,5,6]. The light sources, optical paths, receiver chains, and timing control units for laser ranging, beacon tracking, communication, and imaging are still often configured separately. This leads to duplicated optical components, increased alignment complexity, and higher size, weight, and power consumption, which are unfavorable for spaceborne or maneuvering platforms with strict size, weight, and power (SWaP) constraints. In addition, previous studies have mainly focused on single-function performance verification, such as laser communication rate, ranging accuracy, or imaging quality, while the cooperative operation of imaging, ranging, and communication under a unified optical architecture has not been sufficiently validated.

In complex near-ground and space target observation scenarios, atmospheric haze, turbulence, and background scattering can further degrade optical imaging quality and reduce target–background separability. Recent studies on haze recognition and mitigation have shown that haze-induced degradation is an important factor affecting reliable optical and remote sensing image interpretation [11]. Therefore, it is necessary to introduce polarization imaging into an integrated optical terminal to provide additional target–background discrimination information under low-contrast atmospheric conditions.

To address these limitations, this work proposes an integrated imaging, ranging, and communication architecture based on fiber phased array beam splitting and coupling. The proposed architecture integrates the ranging laser, communication beacon, and signal light source at the light source level and combines the laser ranging, visible light polarization imaging, and high-speed coherent communication channels through a shared Cassegrain optical system and beam splitting structure. The main contribution of this work lies not only in the combination of three functions, but also in the coordinated optical path design and ground-based experimental verification of multi-function operation, including visible light polarization imaging under haze conditions, UAV-based dynamic ranging, and 20 Gbps coherent free-space laser communication.

The main contributions of this work are summarized as follows:

First, a fiber phased array beam splitting–coupling architecture is introduced for light source integration. This architecture enables the coordinated use of ranging light, communication beacon light, and communication signal light, thereby reducing the dependence on separated light source modules and improving the compactness of the optical terminal.

Second, a shared Cassegrain optical system with a beam splitting configuration is designed to integrate visible light polarization imaging, pulsed laser ranging, and coherent laser communication within a unified optical architecture. This design reduces duplicated optical channels and improves the feasibility of multi-functional integration under SWaP-constrained platform conditions.

Third, visible light polarization imaging is incorporated into the integrated system to enhance target–background separability in haze and low-contrast environments. Compared with conventional intensity imaging, the polarization channel provides complementary information for target detection and imaging under complex atmospheric conditions.

Fourth, a ground-based experimental validation framework is established to evaluate the cooperative operation of the integrated system. The experiments include polarization imaging at kilometer-level distances, UAV-based dynamic ranging, and 20 Gbps QPSK coherent free-space laser communication, providing experimental support for assessing the applicability of the proposed architecture to space target observation tasks.

## 2. Overall Scheme for Integrated Imaging, Ranging, and Communication Technology

### 2.1. System Composition and Working Flow

The integrated laser imaging, ranging, and communication system proposed in this study adopts a modular design concept and consists of four functionally coupled subsystems: a laser ranging and communication beacon subsystem, information imaging and communication transmission subsystem, laser detection and beacon and signal light source subsystem, and target acquisition, pointing, and tracking subsystem.

**Figure 1:**
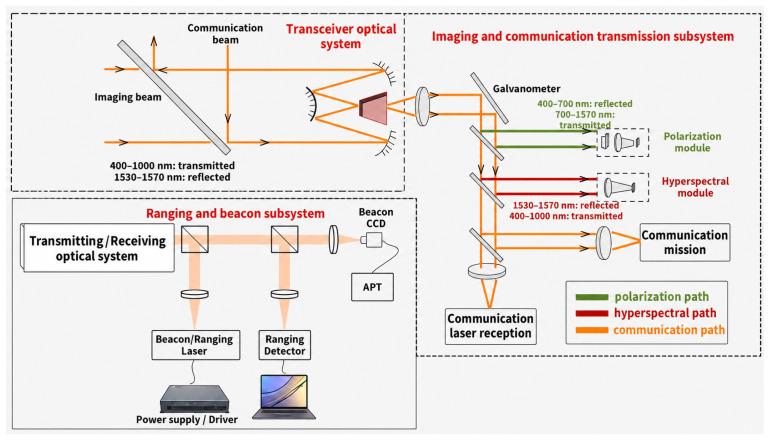
System block diagram.

Among them, the laser communication beacon and laser ranging subsystem is composed of a beacon and ranging transmitting subsystem and a beacon and ranging receiving subsystem. The laser ranging and communication beacon subsystem shares one optical system; the information imaging and communication transmission subsystem shares another optical system; and the laser ranging and beacon subsystem shares the same laser source with the signal light source. The overall structure is shown in Figure 1.

The main functions of each subsystem are as follows:

(1) The laser ranging unit of the laser ranging and communication beacon subsystem transmits a 1064 nm active laser to scan spatial targets and receives reflected light from the targets for ranging. The communication beacon unit receives reflected light from the target or beacon light from other optical terminals to complete coarse tracking for laser communication.
(2) The imaging unit of the information imaging and communication transmission subsystem performs polarization imaging under coarse tracking. The polarization imaging waveband is 400–700 nm; the aperture, focal length, and field of view are determined by the application environment. The communication transmission unit transmits signal light and receives signal light from other optical terminals simultaneously to achieve fine tracking and transmission for laser communication.
(3) The laser ranging and beacon signal light source adopts optical fiber phased array beam splitting and coupling technology to realize multi-wavelength, high-power, and high-stability light source integration for laser ranging, beacon, and signal light, thus achieving system miniaturization and lightweight design.
(4) The target acquisition, pointing, and tracking subsystem can realize guiding and pointing, acquisition and pointing, coarse tracking and fine tracking, and finally complete laser communication.

**Figure 2:**
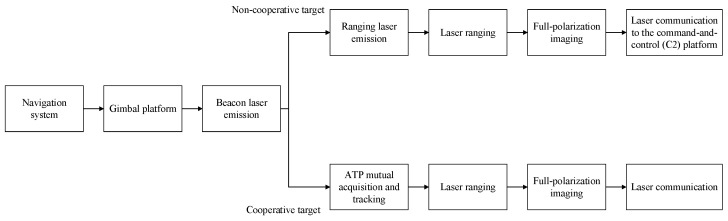
Operational flowchart of laser ranging, imaging, and communication system.

The working process of the laser ranging, imaging, and communication system is shown in Figure 2. First, the navigation system determines the target and sends information to the turntable. For cooperative targets, the local unit transmits a beacon/ranging laser, and the opposite unit also transmits a beacon/ranging laser. The two parties realize acquisition and tracking through the APT (Alignment, Acquisition, Tracking) system, perform visible light linear polarization imaging simultaneously, process the received ranging light to realize laser ranging, and transmit communication laser for data exchange. For non-cooperative targets, ranging laser is transmitted, acquisition and tracking are realized through the local APT system, ranging is performed using reflected light, visible light linear polarization imaging of the target is carried out, and the obtained information is transmitted back to the local unit via the laser communication system [12].

The integrated laser imaging, ranging and communication system studied in this paper is mainly oriented to situation awareness of spatial non-cooperative targets. Typical application scenarios include precise orbit determination and feature recognition of space debris, on-orbit state assessment of failed satellites, and close-range observation of non-cooperative targets. Such tasks put forward the following core requirements for the detection system:

Multi-function integration: synchronously obtain target distance, visual image and characteristic polarization information on a single platform, and establish a high-speed data return link.

Miniaturization, lightweight, and high reliability: adapt to the strict constraints on payload volume, weight, and power (SWaP) of satellite-borne or space maneuvering platforms and possess space environment adaptability.

Dynamic adaptability: realize stable acquisition, tracking, and measurement for non-cooperative targets with certain relative velocities.

### 2.2. Laser Communication Beacon Light and Laser Ranging Receiving Subsystem

**Figure 3:**
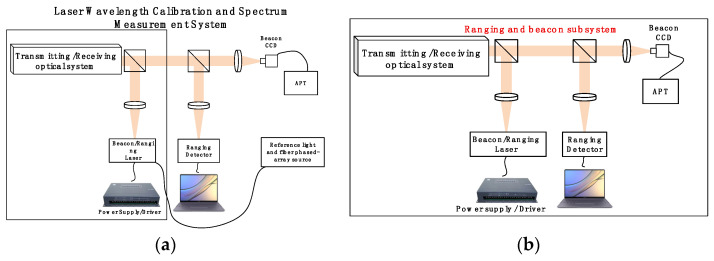
Schematic diagrams of the laser ranging and communication beacon subsystem: (**a**) transmitter and light source integration branch; (**b**) receiving, tracking, and ranging processing branch.

The laser communication beacon light and laser ranging receiving subsystem consists of an optical fiber amplifier, a modulator, transmission fiber, a collimating and beam expander, and a 1064 nm fiber laser, as shown in Figure 3a. The fiber phased array light source can be split and amplified in multiple channels through a coupler, which not only realizes high-power laser output but also provides reference light as the local oscillator light for coherent reception in the communication and ranging systems. Driven and modulated, the fiber phased array light source emits laser pulses through the collimation and beam expansion of the optical system for ranging and imaging.

Although the two optical layouts share a similar beam-expansion and beam splitting structure, they correspond to different functional branches. Figure 3a emphasizes the transmitter and light source integration path, whereas Figure 3b emphasizes the receiving, tracking, and ranging processing path.

The ranging laser is pulsed light, while the communication beacon light is continuous wave light. The repetition frequency of the transmitted laser is 1 kHz–2 kHz, and the operating frequency of the beacon-receiving CCD tracking camera is 50 Hz with an exposure time of 2 ms. Within the exposure time, 2–4 pulse signals are received, enabling target tracking.

### 2.3. Laser Ranging and Communication Beacon Subsystem

The design of the communication subsystem achieves a maximum communication rate of 20 Gbps with a bit error rate (BER) no higher than 1 × 10^−7^. This performance is realized through high-order QPSK/M-QAM modulation technology and a coherent detection scheme, and the signal quality is ensured by EDFA amplification and an adaptive optical system. Within the 1 km ground verification experiment, the system was used to evaluate the high-speed coherent communication capability under a near-ground free-space path. Together with the link budget analysis, the result provides a feasibility reference for longer-distance optical communication under the assumed receiver sensitivity and optical path parameters.

The ranging subsystem is required to achieve a ranging accuracy of no more than 2 m in dynamic scenarios. This indicator is jointly guaranteed by the 1064 nm pulsed laser ranging scheme, a high-precision timing system, and distance gate control technology. The maximum operating distance of the system is not less than 10 km in an equivalent ground environment. Relying on a high-power fiber laser and a large-aperture receiving optical system, it can support multi-mode ranging of cooperative and non-cooperative targets, meeting the needs of orbit refinement and close-range early warning tasks.

The laser ranging and communication beacon subsystem consists of an optical system, a beam splitter, a laser ranging detector, an attenuation sheet, a coarse beacon-receiving CCD, a counter, and a laser ranging processing system [13], as shown in Figure 3b. The laser signal emitted by the laser hits the target through the atmosphere. For cooperative units, part of the optical signal passes through the beacon optical system, hits the beacon-receiving CCD through the beam splitter for reception, and captures and tracks the laser in preparation for laser communication, and the other part is reflected back and received by the laser ranging optical system for counting to measure the distance; for non-cooperative targets, communication is not required, and the reflected laser is directly used for ranging. To receive the pulsed laser spot emitted by the coarse beacon, a pulsed laser with a repetition frequency greater than 2 kHz is considered at the receiving end according to the operating frequency of the CCD (25 Hz), so that the CCD can continuously receive the laser spot. Then, considering the impact of the high instantaneous power of the aforementioned pulsed laser on the detection device, adding a reasonable attenuation device can realize the integration of laser ranging and laser communication coarse beacon. Considering that the field of view of the laser communication signal light receiving and visible light linear polarization imaging detection subsystem is small, it is planned to adopt a combination of general survey and detailed survey detection methods, that is, to use the large field of view of the laser communication beacon light and laser ranging receiving subsystem for general survey detection, search for targets within a large field of view, then stably track the targets with APT technology, and then perform visible light linear polarization imaging-based detailed survey detection [14].

The ranging laser is pulsed light, while the communication beacon light is continuous light. The repetition frequency of the transmitted laser is 1 kHz–2 kHz, and the operating frequency of the beacon-receiving CCD tracking camera is 50 Hz with an exposure time of 2 ms. Within the exposure time, 2–4 pulse signals are received to realize target tracking [15].

To cover the short-range ranging of typical low-Earth orbit debris, the accuracy needs to meet the requirements of orbit element refinement and close-range operation safety distance early warning. To meet this requirement, an operating distance of 100 km and a meter-level accuracy are required, which is verified for feasibility through ground equivalent experiments in this paper.

A high-precision pulsed laser ranging system and optical path integration design are adopted. A 1064 nm pulsed laser is used, combined with a high-precision time counter and distance gating technology, to realize accurate distance measurement of cooperative and non-cooperative targets. By sharing the optical antenna and transceiver path between the ranging light and the communication beacon light, combined with a compound-axis APT system, the system is designed to achieve meter-level ranging performance under dynamic scenarios, while the UAV-based field experiment in Section 4.3 verifies the acquisition, tracking, and ranging capability of the integrated system under the tested conditions.

The communication beacon subsystem adopts a high-rate coherent laser communication link and an integrated optical terminal design. The system uses high-order modulation formats such as QPSK and M-QAM and coherent reception technology, which supports high-speed coherent signal transmission in the ground verification experiment. In the 1 km free-space communication test, the system achieved 20 Gbps QPSK signal transmission with a bit error rate on the order of 10^−7^. The link budget analysis further indicates that, under the assumed receiver sensitivity and optical path parameters, the proposed configuration can provide a positive power margin for a 10 km-class link. The satellite trajectory optical simulator technology can effectively simulate the relative movement of space targets on the ground, which provides an important experimental method for the dynamic APT performance test and verification of the system [16], laying a foundation for long-distance, high-rate space data return.

### 2.4. Information Imaging and Communication Transmission Subsystem

In terms of the imaging subsystem, the system is designed with an operating distance of 5 km, adopting a Cassegrain optical system with an aperture of no less than 200 mm and a focal plane division polarization imaging technology to ensure high-quality image acquisition within the typical close range of low-Earth orbit debris. Polarization imaging technology is introduced to provide complementary target–background contrast under haze and low-contrast atmospheric conditions. The representative field experiments in Section 4.2 further quantify the contrast improvement obtained by the polarization channel. The imaging waveband covers the 400–700 nm visible light range, and effective extraction of target reflection and polarization characteristics is achieved through a beam splitting structure and a filter system.

**Figure 4:**
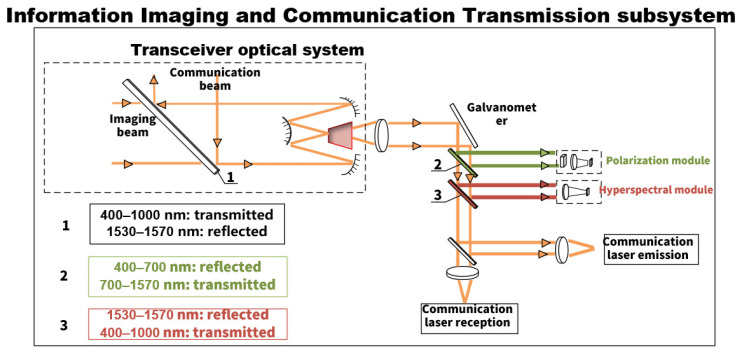
Imagsing and communication transmission subsystem.

The information imaging and communication transmission subsystem consists of a laser communication transmitting system, a laser communication receiving system, and an imaging system, including a Cassegrain telescope optical system, a beam splitter, a galvanometer, an imaging CCD, a communication CCD, a communication laser source, an APT processing system, and a turntable. To meet the long-distance working requirements of imaging, communication, and ranging simultaneously, a large-aperture optical system must be adopted to collect sufficient optical signals. The Cassegrain optical system has the advantages of long focal length, small volume, aberration correction, and polarization imaging integration. For the resolution improvement of the optical system, the binary phase filter technology can effectively narrow the point spread function (PSF) core to achieve transverse super-resolution, and the Strehl ratio limit corresponding to the core size can provide an important theoretical basis for the resolution and image quality balance design of the imaging system [17,18]. The structural design is shown in Figure 4. The turntable, APT system, and Cassegrain optical system of laser communication are used for transmitting and receiving communication light, and the imaging system is installed in the laser communication Cassegrain telescope optical system through a beam splitting method. Although this reduces the field of view of imaging, the tracking and stabilization functions of the turntable and APT system can better achieve rapid acquisition and stable tracking of moving targets, realizing the combination of the laser communication and imaging subsystems.

It should be noted that Figure 1 presents the system-level block diagram of the complete integrated imaging, ranging, and communication architecture, whereas Figure 4 provides a detailed optical layout of the information imaging and communication transmission subsystem. Therefore, the two figures are not duplicated; Figure 1 describes the overall functional coupling among subsystems, while Figure 4 focuses on the internal optical path, beam splitting structure, and receiver arrangement of the imaging and communication module.

The Cassegrain optical system and beam splitting devices support communication light transmission and reception while also enabling visible light polarization imaging through the shared optical path. The large-field-of-view target observation camera realizes general survey, and the small-field-of-view Cassegrain system realizes detailed survey with stable imaging [9].

## 3. Mathematical Model of the Ranging, Imaging, and Communication System

### 3.1. Visible Light Polarization Imaging Model

This chapter aims to clarify how to effectively verify and evaluate the core performance of the system in the space long-distance environment through ground-equivalent distance experiments. By quantitatively analyzing the physical differences between the space and ground links, the equivalent conversion relationship of key parameters is established so as to provide a constrained basis for assessing the applicability of the ground experimental results to space target observation scenarios.

#### 3.1.1. Illuminance Analysis of Visible Light Polarization Imaging

As an electromagnetic wave, the vibration direction of the electric field vector E of light is asymmetric with the propagation of light, which is called the polarization of light [19]. The polarization state of light waves is usually described by the Stokes vector (S_0_, S_1_, S_2_, S_3_)ᵀ, which can also be written as (I, Q, U, V)ᵀ, where I represents the total light intensity, Q and U represent the linear polarization components at different angles, and V represents the circular polarization component [20].

(1) $$S=\left[\begin{array}{l} S_{0}\\ S_{1}\\ S_{2}\\ S_{3} \end{array}\right]=\left[\begin{array}{l} I\\ Q\\ U\\ V \end{array}\right]=\left[\begin{array}{l} I_{0}+I_{90}\\ I_{0}-I_{90}\\ I_{45}-I_{135}\\ I_{rc}-I_{lc} \end{array}\right]$$

The present experiment focuses on linear polarization imaging using the 0°, 45°, 90°, and 135° channels of the division-of-focal-plane detector. Therefore, the polarization-derived images and contrast analysis in this work are based mainly on the linear polarization components, while the circular polarization component is listed only for the completeness of the Stokes representation.

In the formula, $I_{0}$ and $I_{90}$ are the light intensities in the 0° and 90° polarization directions, respectively; $I_{45}$ and $I_{135}$ are the light intensities in the 45° and 135° polarization directions, respectively; $I_{rc}$ and $I_{lc}$ are the light intensities of right-handed and left-handed circularly polarized light, respectively. The change in light polarization parameters can well describe the change of light polarization state, among which the degree of polarization (*DOP*) is one of the important parameters in light polarization parameters. The degree of polarization is defined as the ratio of the polarized light component to the total light wave component, and its expression is:

(2) $$DOP=\frac{\sqrt{Q^{2}+U^{2}+V^{2}}}{I}$$

The angle of polarization (*AOP*) is also an important parameter among the light polarization parameters. It is defined as the phase difference between the two radiation components, and its expression is:

(3) $$AOP=\frac{1}{2}\arctan\left(\frac{U}{Q}\right)$$

Through the above calculation formula of the Stokes vector, the polarization parameters can be further obtained so as to derive the polarization information of the detected target. For the quantitative analysis of the polarization characteristics of space target materials, the polarization bidirectional reflection distribution function (pBRDF) model considering the masking effect can effectively invert the physical parameters of the target surface (refractive index, roughness, etc.), which provides a more accurate theoretical support for the design of the polarization imaging system [20].

Polarization imaging can simultaneously acquire two-dimensional spatial information and polarization information of the target, capture the difference in polarization characteristics between the target and the background, and has the advantages of high contrast and high sensitivity. Combined with the spatially modulated interferometric spectral imaging technology represented by SMIFTS [21], which can realize the simultaneous measurement of all infrared spectral channels under a wide field of view, the multi-dimensional information acquisition capability of space targets including polarization, spatial, and spectral characteristics can be further improved. Therefore, polarization detection has attracted much attention in various fields. At present, the widely used polarization detection and imaging methods include time-division polarization detection, split-aperture polarization detection, split-amplitude polarization detection, and focal plane division polarization detection. Since these methods have their own advantages and disadvantages, they can be reasonably selected in different scenarios. This study adopts the focal plane division polarization detection [22].

**Figure 5:**
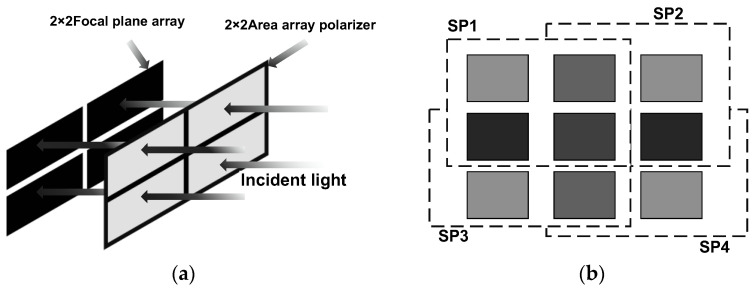
Schematic diagram of the focal plane division polarization imaging principle: (**a**) integration of the detector and micro-polarizer array; (**b**) polarization imaging processing.

The focal plane division method integrates polarization elements onto the focal plane, where one pixel on the focal plane corresponds to one micro-polarization element. Figure 5 below shows a schematic diagram of the structure of the focal plane division polarization imaging detector. A layer of micro-nano structure polarization array is processed or pasted on the focal plane of the imaging detector, with every four pixels used as a group, each sensitive to polarization vectors in different directions. During polarization imaging calculation, the response of the current pixel and its surrounding pixels is used to directly or indirectly obtain the polarization components or polarization state of the pixel in different directions, and then the Stokes vector is calculated to complete the polarization imaging calculation. The focal plane division method can simultaneously acquire polarization components or polarization states of incident light in different directions, so it can image both static and dynamic scenes, and has a compact structure and small volume [23].

The laser-irradiated optical imaging system consists of two parts: a visible light polarization imaging unit and a short-wave infrared active–passive composite imaging unit. Among them, the visible light polarization imaging unit includes three parts: a pre-installed visible light telescope module, a visible light polarization imaging module, and a visible light image acquisition module. The parallel light obtained through the pre-installed visible light telescope module passes through a beam splitter, and the transmitted light enters the visible light polarization imaging module. A visible light focal plane division detector is used to simultaneously acquire polarization information at 0°, 45°, 90°, and 135°, and the polarization information is visually processed through the image acquisition module and displayed on the screen in real time [24].

Because the micro-polarizer array in division-of-focal-plane polarization imaging inevitably introduces spatial sampling loss, polarization super-resolution and restoration methods have been investigated to recover lost spatial information and improve polarization image quality [25].

#### 3.1.2. Illuminance Analysis

During polarization detection, due to the shooting of dynamic targets, the time-division polarization imaging method cannot be adopted, and focal plane division imaging is required. Generally, four polarization states need to be acquired, and polarization imaging needs to consider whether the image plane illuminance can meet the minimum response illuminance requirement of the detector. It is necessary to judge whether the illuminance of the target reaching the image plane is greater than the minimum response illuminance of the detector so as to determine whether the F-number design of the optical system is reasonable.

Spectral irradiance is a radiometric quantity, used to describe the optical power received per unit area and per unit wavelength interval, and it is commonly used in optical measurement and radiometric analysis [26]. Within a small wavelength interval dλ near the wavelength λ, the conversion formula between luminous flux $d\phi_{v}\left(\lambda \right)$ and radiant flux $\phi_{e}\left(\lambda \right)$ is as follows:

(4) $$d\phi_{v}\left(\lambda \right)=K_{m}V\left(\lambda \right)\phi_{e}\left(\lambda \right)d\lambda$$

In the formula: $K_{m}=683lm/W$ $V\left(\lambda \right)=1$ is the absolute spectral efficiency value of monochromatic light with wavelength λ=555nm under photopic conditions. Assuming the bandwidth of the filter is Δλ=0.02 μm, and the reflectivity *γ* of the ground and plants is 0.2, the luminance when the target and background radiate into the space as indirect radiators is:

(5) $$L=\frac{683\cdot \gamma \cdot \Delta \lambda \cdot E_{0\lambda }}{2\pi }=0.435\cdot E_{0\lambda }$$

At this time, the central illuminance of the image plane is:

(6) $$E_{0}=\tau \cdot T\cdot \pi \cdot L\cdot \sin^{2}U$$

In the formula: τ is the atmospheric transmittance; T is the transmittance of the optical system, taken as 0.3; L is the luminance of the target; and U is the object-side aperture angle.

The main factor affecting atmospheric transmittance is atmospheric scattering. According to previous studies, when the visibility is $V_{m}=10\mathrm{km}$, the relationship between transmittance, wavelength, and detection distance l is:

(7) $$\tau (\lambda )=\exp\left[-\frac{3.91}{10}{\left(\frac{0.55}{\lambda }\right)}^{1.3}\cdot l\right]=\exp\left(-\frac{0.1794\cdot l}{\lambda^{1.3}}\right)$$

Then, the formula for the image plane illuminance of the imaging system becomes:

(8) $$E_{0}=\exp\left(-\frac{0.1794\cdot l}{\lambda^{1.3}}\right)\cdot T\cdot \pi \cdot L\cdot \sin^{2}U$$

The minimum detection illuminance of the CCD is $E_{\min0}=0.2\mathrm{lx}$ under the condition of *F* = 1.4, and the minimum detection illuminance of the optical system is $E_{\min}={\left(\frac{F}{1.4}\right)}^{2}E_{\min0}$. When $E_{0}\geq E_{\min}$, the target can be detected.

In hazy weather, the aerosol particles in the haze have a strong scattering and absorption effect on visible light, resulting in the spectral distribution of ground spectral irradiance being biased towards cool tones and the overall irradiance dropping sharply compared with sunny days, about 100–500 lx.

The relationship between the object-side aperture angle sin U and the F-number of the optical system is:

(9) $$sin\;U\approx \frac{1}{2F}$$

Substitute *T*, *L*, and sinU into Equation (11); combined with the inequality $E_{0}\geq E_{\min}$, we can get:

(10) $$\exp\left(-\frac{0.1794l}{\lambda^{1.3}}\right)\cdot 0.3\cdot \pi \cdot 0.435.E_{0\lambda }\cdot {\left(\frac{1}{2F}\right)}^{2}\geq {\left(\frac{F}{1.4}\right)}^{2}\cdot 0.2$$

Substitute the F-number and $E_{0\lambda }$ into it, and the range of detection distance can be obtained.

After calculation, in a hazy environment, the optical system can realize target detection within the range of 3–5 km in the entire visible-to-near-infrared band.

### 3.2. Ranging Energy Model and Analysis

To evaluate the performance potential of the integrated system in the space environment, a theoretical mapping model from ground experiments to space applications is established. Based on the energy equivalence principle, the performance measured at a ground distance L is equivalent to that at a distance Req in free space without atmosphere, where:

(11) $$R_{\mathrm{eq}}=\frac{L}{\sqrt{T_{\mathrm{atm}}(L)\cdot L_{\mathrm{turb}}}}$$

where $L_{\mathrm{turb}}$ is the equivalent attenuation factor caused by turbulence and $T_{\mathrm{atm}}(L)$ is the atmospheric transmittance function. At a ground test distance of 5 km, the atmospheric attenuation and optical path degradation provide a constrained near-ground validation condition for evaluating target acquisition, tracking, and imaging feasibility. This scaled experiment is used as a feasibility verification rather than a complete reproduction of the space target observation environment.

In the ground scenario, the attenuation equation for laser irradiating a diffuse small target before entering the optical system is:

(12) $$\frac{W_{r}}{W_{t}}=\frac{2A_{r}\delta }{\pi^{2}\theta_{t}^{2}R^{4}}T_{t}T_{r}{T_{q}}^{2}\rho \eta_{p}$$

where $W_{r}$ is the received pulse energy; $W_{t}$ is the transmitted pulse peak energy; $\theta_{t}$ is the laser beam divergence angle; R is the operating distance; Ar is the aperture area of the receiving optical antenna;  δ is the scattering cross-section of the target; ρ is the target reflectivity; $\eta_{p}$ is the detector quantum efficiency; $T_{t}$ is the transmittance of the transmitting optical system; and $\;T_{r}$ is the transmittance of the receiving optical system.

For a random photon flux, the probability of detecting k photons follows a Poisson distribution:

(13) $$p_{k}\left(N\right)=\frac{N^{k}\exp(-N)}{K!}$$

The probability of obtaining more than one photoelectron can be calculated as:

(14) $$P=1-e^{-n_{0}}$$

where $n_{0}$ is the number of acquired photons, $n_{0}=W_{r}/W_{p}\;$, and $W_{p}$ is the single-photon energy, $W_{p\;}=h_{c}/\lambda$.

In this ground laser ranging experiment, the set detection distance is 10 km, and a standard target with a size of 5 cm is used to simulate the ranging demand for small-sized targets in the ground scenario. Although there are interferences from natural background light (e.g., sunlight, ambient scattered light) and atmospheric backscattering in the ground environment, the influence of background light and atmospheric scattering can be effectively suppressed by adding a narrowband filtering module and a light-shielding structure in the experimental setup. In this case, the system detection noise mainly comes from the dark count of the laser detector itself, which is tested to be 3000 c/s @ 20% QE (quantum efficiency). Combined with the experimental set ranging distance of 10 km, the round-trip time of the laser signal is about 66.7 μs. In the actual detection process, only a range gate width of 1 μs is needed to accurately capture the target reflection signal. The noise generated by the detector dark count within this narrow gate width is further reduced, and its influence on the accuracy of ranging data can be neglected.

In the ranging subsystem, the signal power received by the detector is:

(15) $$P_{r}=P_{t}\cdot \frac{K\cdot A_{r}\cdot \delta \cdot \rho \cdot \cos\phi }{\theta_{t}^{2}\cdot R^{2}}T_{t}\cdot T_{r}\cdot T_{a}$$

where $P_{t}$ is the transmitting power of the transmitting unit; K is the light speed profile function, set as K = 1; $A_{r}$ is the receiving area with an aperture diameter of 200 mm; δ is the target reflection area with a diameter of 100 mm; ρ is the target reflectivity, set as ρ = 0.2; θ is the angle between the target reflection normal and the transmitted beam; cosϕ=1 is the transmitting beam divergence angle, set as $\theta_{t}=1\;\mathrm{mrad}$; R is the distance between the space target and the detector; $T_{t}$ is the transmittance of the transmitting optical system, set as $T_{t}=0.33$; $T_{r}$ is the transmittance of the receiving optical system, set as $T_{r}=0.8$; and $T_{a}$ is the single-layer transmittance under good atmospheric conditions, set as $T_{a}=0.7$.

(16) $$P_{r}=P_{t}\cdot \frac{K\cdot A_{r}\cdot \delta \cdot \rho \cdot \cos\phi }{\theta_{t}^{2}\cdot R^{2}}T_{t}\cdot T_{r}\cdot T_{a}=P_{t}\cdot \frac{1.82\times 10^{7}}{10^{14}}=10^{-5}W$$

When the operating distance is 10 km, the calculated result is consistent with the empirical distance–power curve. Under certain conditions, the required transmitting power of a laser ranging system using a 1.06 μm laser is greater than 54.88 W.

### 3.3. Laser Communication Energy Model and Analysis

The laser communication equation is transformed from the electromagnetic wave propagation equation [27]. Under the diffraction-limited condition, the laser communication equation is:

(17) $$P_{r}=P_{t}\cdot G_{t}\cdot \eta_{ot}\cdot L_{s}\cdot \eta_{s}\cdot L_{APT}\cdot G_{r}\cdot \eta_{or}$$

where $P_{r}$ is the received power; $P_{t}$ is the transmitted power; $G_{t}$ is the gain of the transmitting optical antenna; $\eta_{ot}$ is the efficiency of the transmitting optical unit; $L_{s}$ is the link attenuation caused by free space; $\eta_{s}$ is the power loss caused by the channel (approximately 1 for free-space laser communication, with varying degrees of attenuation for atmospheric and seawater channels); $L_{APT}$ is the power loss caused by APT pointing mismatch; $G_{r}$ is the gain of the receiving optical antenna; and $\eta_{or}$ is the efficiency (transmittance) of the receiving optical system.

The vacuum laser communication propagation equation can be derived from Equation (17):

(18) $$P_{r,\mathrm{vac}}=P_{t}\cdot \eta_{t}\cdot \eta_{r}\cdot G_{t}\cdot G_{r}\cdot {\left(\frac{\lambda }{4\pi R}\right)}^{2}$$

where $P_{t}$ is the transmitted power, λ is the laser wavelength, $\eta_{t}$ and $\eta_{r}$ are the transmitting and receiving optical efficiencies, $G_{t}$ and $G_{r}$ are the antenna gains, and R is the transmission distance.

For the laser communication unit, using power budget analysis, as shown in Table 1 the equivalent communication distance can be better than 10 km. At a communication rate of 20 Gbps, semiconductor external modulation technology and EDFA high-power amplification (3 W) are adopted; a margin of more than 3 dB is reserved in the design to further improve channel adaptability. The APD limit sensitivity is −20 dBm at BER = 10^−7^ and 20 Gbps [28].

The link budget result indicates that, under the assumed APD receiver sensitivity, transmitting power, optical efficiency, atmospheric loss, and receiving aperture, the proposed optical configuration has sufficient power margin for a 10 km-class communication link. This analysis is used as a system-level feasibility estimate rather than a direct experimental verification of the long-distance communication performance.

## 4. Ground-Based Experimental Validation of the Integrated Imaging, Ranging, and Communication System

Visible light polarization imaging was used to evaluate the imaging capability of the integrated platform under complex near-ground atmospheric conditions. Compared with conventional intensity imaging, polarization imaging can exploit the polarization difference between the target and the background, thereby improving target–background separability when image contrast is degraded by haze, turbulence, or background interference. In this section, field imaging experiments are conducted to verify the effectiveness of the polarization imaging module. The results also provide experimental support for assessing the applicability of the proposed system to long-distance observation and space target monitoring, together with the equivalence model established in the previous section.

### 4.1. Integrated Device for Space Target Imaging, Ranging, and Communication

To verify the technical feasibility and design rationality of the laser communication–ranging–imaging integrated system scheme, this study designs and carries out indoor simulation verification experiments and outdoor physical verification experiments, which specifically include two types of core verification contents: first, through the collaborative test experiment of the laser ranging and imaging system, verify the stability of the laser communication beacon light link and the functional effectiveness of the laser ranging receiving subsystem; second, through the joint simulation experiment of the laser communication signal light transceiver link and the imaging detection subsystem, verify the compatibility and cooperative work feasibility of the laser communication function and imaging function at the hardware integration and signal timing matching levels.

The above experiments provide key practical support for the engineering implementation of the laser communication–ranging–imaging integrated technology, and their verification conclusions have important guiding significance and reference value for the subsequent system performance testing and product R&D in the field environment.

**Figure 6:**
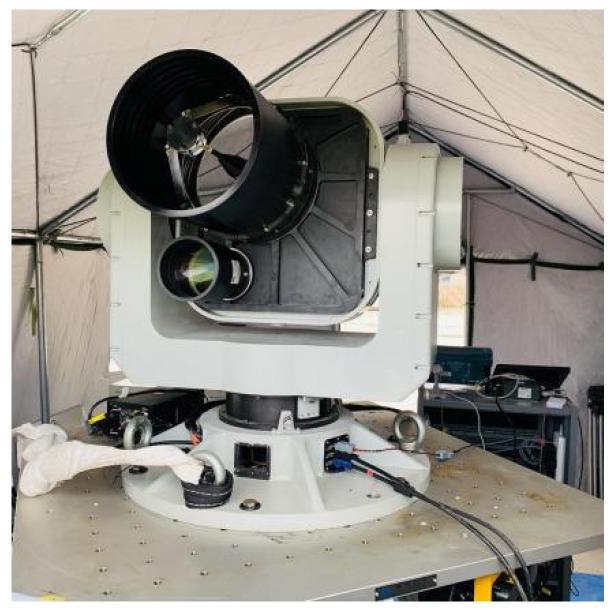
Physical prototype of the integrated experimental device for space target imaging, ranging, and communication.

Based on the overall system technical scheme and core mathematical model, this study further develop an integrated experimental device for space target imaging–ranging–communication, as shown in Figure 6. In addition to the functions of laser ranging and laser communication for space targets, the device also integrates a visible light polarization imaging module, which can realize multi-dimensional information detection of targets.

### 4.2. Visible Light Polarization Imaging Verification

Visible light polarization imaging experiments were conducted to evaluate the detection and imaging capability of the proposed integrated system under complex near-ground atmospheric conditions. This section first establishes the optical system equivalence between the ground-based experimental configuration and the intended long-distance observation scenario. Then, representative intensity and polarization imaging results are compared to assess the target–background separability, image contrast, and the complementary information provided by the polarization channel.

#### 4.2.1. Optical System Equivalence

In this paper, a 1:10 equivalent scaling ratio is adopted to construct a high-tolerance and high-precision hardware-in-the-loop simulation system for angle and range measurement of low-Earth orbit targets, and field tests on angle and range measurement accuracy are carried out using this system. The developed hardware-in-the-loop simulation system is used to track and image a 5 cm target carried by an unmanned aerial vehicle at a distance of 5 km. The main reasons are as follows:

When the imaging distance is no more than 5 km, the influence of atmospheric fluctuations on the imaging of the optical system is small, so the ground demonstration can provide scaled verification of the angular size relationship, target acquisition, and imaging feasibility under a controllable near-ground atmospheric path.

**Figure 7:**
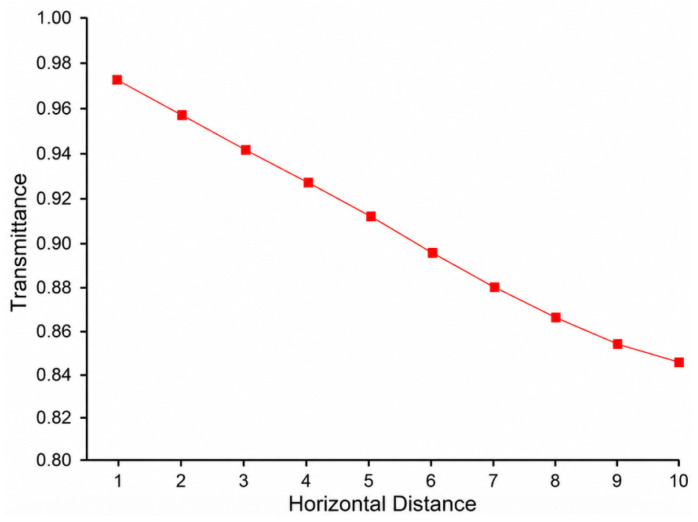
Relationship between optical system transmittance in the atmosphere and detection distance.

According to the analysis by the atmospheric transmission simulation software MODTRAN 5, the atmospheric transmittance of the optical system is greater than 90% within a detection distance of 5 km in clear weather, as shown in Figure 7; when the ground detection distance exceeds 5 km, the increased near-ground atmospheric attenuation may introduce additional uncertainty into the scaled verification. Therefore, a 5 km test distance was selected to maintain a controllable atmospheric path while satisfying the angular size equivalence requirement.

According to the feasibility analysis of space-based system indicators, the aperture of its optical system reaches 1.2 m, so large-aperture reflectors must be adopted. The 1:10 scaling system can meet the requirements of principle verification.

The influence of equivalent scaling on detection distance and target size was further analyzed as follows.

For observing a target with a size of 1 m at a distance of 1000 km, the detection capability is 1 m@1000 km.

Due to the influence of detection distance on the atmospheric transmittance of the optical system and the limitation of optical system cost, the detection distance of the equivalent scaling target angle and range measurement field demonstration and verification platform is set to 5 km.

For observing a target with a size of 1 m at a distance of 1000 km, after 1:10 scaling, it is equivalent to observing a target with a size of 1 m at a distance of 100 km.

(19) $$\frac{1\;m}{100\;\mathrm{km}}=\frac{5\;\mathrm{cm}}{5\;\mathrm{km}}\;$$

Therefore, after equivalent conversion, the 1:10 equivalent scaling target angle and range measurement field demonstration and verification platform needs to observe a target with a size of 5 cm at a distance of 5 km.

The scaled ground experiment is intended for principle verification rather than full reproduction of the space target observation environment. The main equivalence assumptions and validity constraints are summarized in Table 2.

Therefore, the ground experiment provides scaled verification of optical resolution, target acquisition, tracking, and ranging capability under a controllable near-ground atmospheric path, but it should not be interpreted as a complete reproduction of the space target observation environment.

#### 4.2.2. Detection and Imaging Results

For quantitative comparison, the target region and the adjacent background region were manually selected at the same spatial positions in the intensity, polarization, and fused images. The contrast was calculated as

(20) C=It−IbIb×100%

where $I_{t}$ and $I_{b}$ denote the mean gray values of the target and background regions, respectively. The same region-selection criterion was used for each image pair to ensure a consistent comparison. Since the field experiments were conducted under natural atmospheric conditions, the reported contrast values are used as representative comparison results rather than exhaustive statistical averages over all weather conditions.

**Figure 8:**
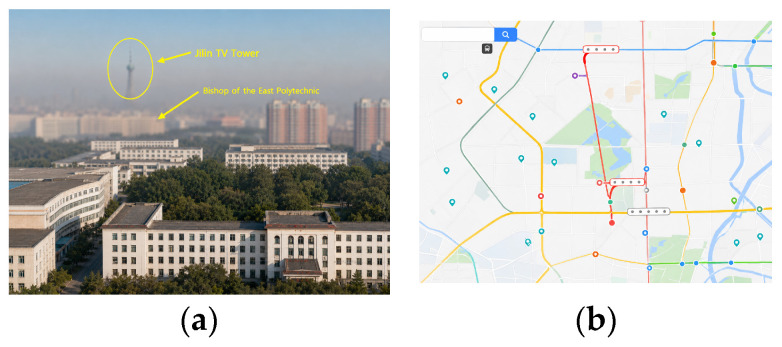
Experimental site for visible light polarization imaging: (**a**) actual view of the test site; (**b**) corresponding location of the test site on the map.

**Figure 9:**
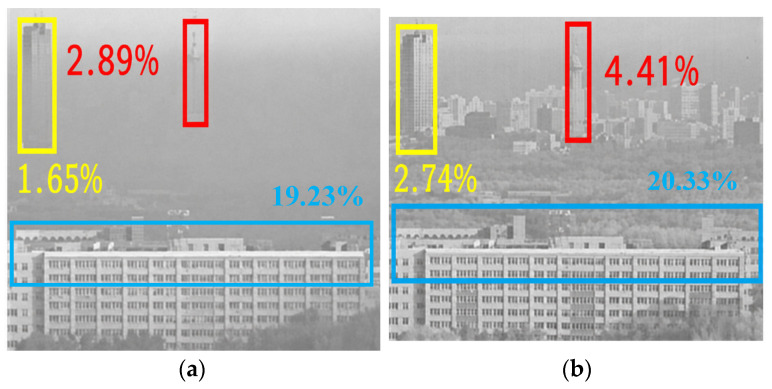
Comparison of imaging results: (**a**) conventional intensity image; (**b**) fused image generated using intensity and polarization-derived information.

The visible light polarization imaging system was used to observe the Jilin TV Tower at a distance of 5 km, as shown in Figure 8. The target contour in the fused image is clear, and the distinction between distant trees (natural targets) and buildings (artificial targets) is obvious. As shown in Figure 9, in the intensity image, the contrast of the high-rise building circled by the yellow frame is 1.65%, which increases to 2.74% in the fused image; in the intensity image, the contrast of the TV tower circled by the red frame is 2.89%, which increases to 4.41% in the fused image; the building circled by the blue frame is a near target, which can be clearly seen in both the intensity image and the fused image.

**Figure 10:**
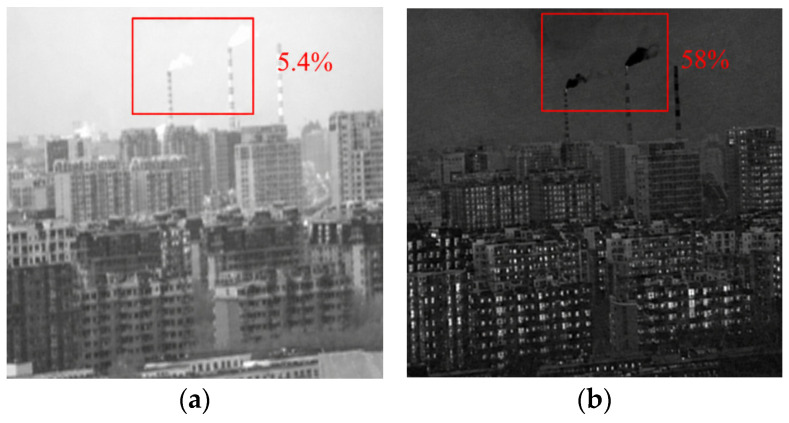
Passive polarization imaging of a haze scene at an imaging distance of L = 3000 m: (**a**) intensity imaging; (**b**) polarization imaging.

Multiple visible light polarization imaging detection experiments were carried out using the developed polarization imaging system. Under haze conditions, a chimney 3 km away was imaged, and the experimental results are shown in Figure 10. In the intensity image, the contrast of the smoke circled by the red frame is 5.4%, while in the polarization image, the contrast of the same target is increased to 58%. In a haze environment, the polarization image provides higher target–background contrast than the corresponding intensity image.

Visible light polarization imaging detection experiments were carried out in summer and winter using the developed polarization imaging system.

**Figure 11:**
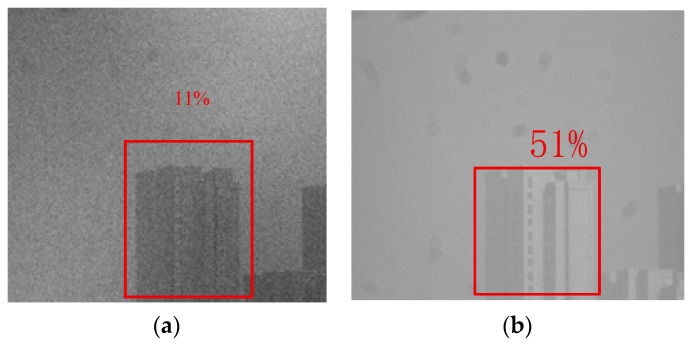
Passive polarization imaging of a summer scene at an imaging distance of L = 1500 m: (**a**) intensity imaging; (**b**) polarization imaging.

As shown in Figure 11, the visible light polarization imaging system was used to image a building 1.5 km away in a summer haze environment. In the intensity image, the contrast of the glass window circled by the red frame is 11%, while in the polarization image, the contrast of the selected target is increased to 51%.

**Figure 12:**
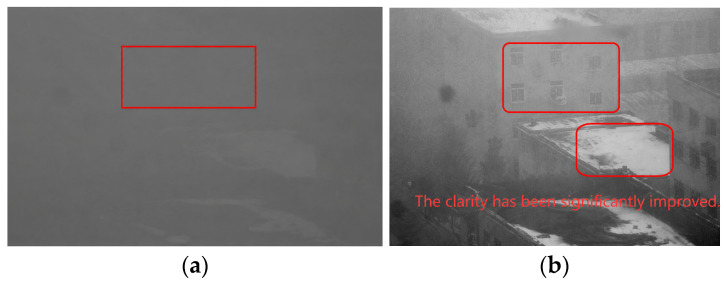
Passive polarization imaging of a winter scene at an imaging distance of L = 1500 m: (**a**) intensity imaging; (**b**) polarization imaging.

As shown in Figure 12, the visible light polarization imaging system was used to image a building 1.5 km away in a winter haze environment. In the intensity image, the contrast of the glass window circled by the red frame is 5%, while in the polarization image, the contrast of the selected target is increased to 49%.

It can be seen from the experimental results in Figure 12 that in the atmospheric environment, the scattering of background light weakens the brightness difference between the target and the background, resulting in low contrast of glass windows and difficulty in clear resolution. Since glass windows are smooth reflecting surfaces, they produce reflected light with high polarization degree. There is a significant difference between the polarization characteristics of atmospheric scattered light and the reflected polarization characteristics of glass windows. This difference in polarization information improves the target contrast, making the glass windows more clearly visible in the polarization image.

It can be seen from multiple groups of experimental images that by utilizing the difference in polarization characteristics between smoke and the background, visible light polarization imaging can improve target–background contrast when conventional intensity contrast is degraded by haze scattering, making the effect of polarization images better than that of intensity images [29]. These results indicate that polarization-derived information can provide complementary target–background contrast under haze and low-visibility conditions, which is beneficial for long-distance target observation when conventional intensity contrast is degraded.

The experiments in this subsection mainly verify the passive visible light polarization imaging capability based on division-of-focal-plane polarization detection. The polarization images were obtained from polarization-dependent intensity information, while the fused images were generated by combining intensity information with polarization-derived features. The representative haze experiments show that polarization-derived information can provide complementary target–background contrast under degraded visibility conditions.

### 4.3. Laser Ranging Results

#### 4.3.1. Distance Measurement Scale Test Platform

A scaled ground demonstration platform was established to verify the ranging, tracking, and acquisition functions of the integrated system under controllable field conditions. The platform was used to evaluate the feasibility of the ranging subsystem and to provide experimental support for the subsequent dynamic ranging test.

On a clear, cloudless night with high visibility, an integrated demonstration was conducted in an open area with low background light in a suburban city to simulate a dark deep-space background. The accurate distance was extracted from the laser ranging data using a distance gate determined based on prior knowledge. The real-time spatial coordinates of both the ranging subsystem and the BeiDou receiver and inertial navigation module mounted on the diffuse reflective small target were determined, and the accurate distance (in centimeters) was further calculated, thus verifying the tracking capability and ranging accuracy. The dynamic measurement capability was verified by simulating the motion of a satellite platform using a six-degree-of-freedom swing table set up under the ranging platform. The relative angular velocity of a low-Earth orbit target relative to the laser ranging system was simulated by controlling the distance between the UAV and the integrated demonstration device, the UAV’s speed, and their positional relationship.

The impact of equivalent scaling on ranging accuracy is analyzed as follows:

The modeled single-shot ranging uncertainty is mainly determined by pulse width, timing accuracy, detector delay, calibration error, and platform motion-induced optical path variation. Therefore, the following uncertainty budget is used to analyze the dominant error sources of the ranging subsystem rather than to represent the statistical distribution of measured ranging errors. The source of optical system error is the residual error after system calibration, which mainly depends on the calibration method and is unrelated to factors such as system focal length and aperture. Therefore, the accuracy of a single ranging measurement remains unchanged before and after scaling, still remaining at 1~2 m.

Table 3 summarizes the modeled uncertainty budget of single-shot laser ranging. It should be noted that this table is used to analyze the dominant error sources of the ranging subsystem rather than to represent the statistical distribution of measured ranging errors.

Therefore, the theoretical uncertainty budget indicates that the scaling of the optical aperture does not significantly change the dominant single-shot ranging error sources. Under the tested dynamic conditions, the ranging results are consistent with the designed meter-level accuracy requirement. However, a more complete statistical evaluation with larger sample numbers and independent reference measurements will be required in future work.

#### 4.3.2. Dynamic Ranging

In the optical equivalence analysis, a 5 cm equivalent target was used to satisfy the scaled angular size relationship. In the dynamic ranging experiment, a 10 cm cooperative target assembly was mounted on the UAV for practical alignment and stable echo acquisition. Therefore, the two target sizes correspond to different verification purposes: the 5 cm target is used for equivalence analysis, whereas the 10 cm target is used for dynamic ranging validation. To test the system’s dynamic ranging performance, a scaled-down integrated demonstration device was built in an outdoor field and used to measure the distance to the 10 cm cooperative target mounted on the UAV.

Before testing, the drone’s positioning system base station, GPS time and frequency receiver, and inertial navigation system were activated, followed by takeoff. The integrated system then powered on to acquire and track the drone. Next, the positioning system and ranging unit on the integrated system were activated, the ranging unit’s distance gate was set, and distance data was acquired. After preparation, the drone began maneuvering. Upon landing, the positioning data was acquired and processed using custom-developed software.

During testing, after the integrated demonstration system performs a self-test upon startup, the tracking camera on the integrated demonstration device completes precise tracking to test the acquisition and tracking capabilities of the ranging signal and the ranging system. The real-time spatial coordinates of the ranging subsystem and the positioning module on the diffuse reflective small target are determined, and the accurate distance between them is further calculated, thereby testing the ranging accuracy of the laser ranging system.

Since the previous field experiment was mainly designed for functional verification rather than exhaustive statistical evaluation, the raw ranging statistics and independent reference uncertainty were not sufficient to support a complete error distribution analysis.

#### 4.3.3. Angular Rate Analysis of System Design

The target motion adaptability of the system is primarily determined by the APT (Acquisition, Pointing, and Tracking) subsystem. Its design specifications are derived from the typical angular velocity of non-cooperative space targets (such as low-Earth orbit debris). The angular tracking bandwidth f_b_ and the maximum stable tracking angular velocity $\omega_{\max}$ of the APT subsystem satisfy:

(21) $$\omega_{\max}\approx 2\pi \cdot f_{b}\cdot \theta_{\max}$$

where $\theta_{\max}$ is the maximum allowable tracking error angle (determined by the detector pixel size and optical focal length). The design parameters of the APT subsystem of this system are: $f_{b}$ ≥ 50 Hz, $\theta_{\max}$ ≤ 25 μrad. Calculated accordingly, the theoretically designed maximum tracking angular velocity of the system is ω = 7.85 mrad/s. At an action distance of R = 100 km, this is equivalent to being able to stably track a target with a transverse velocity of about 785 m/s, completely covering the relative angular velocity of the vast majority of low-Earth orbit targets.

**Figure 13:**
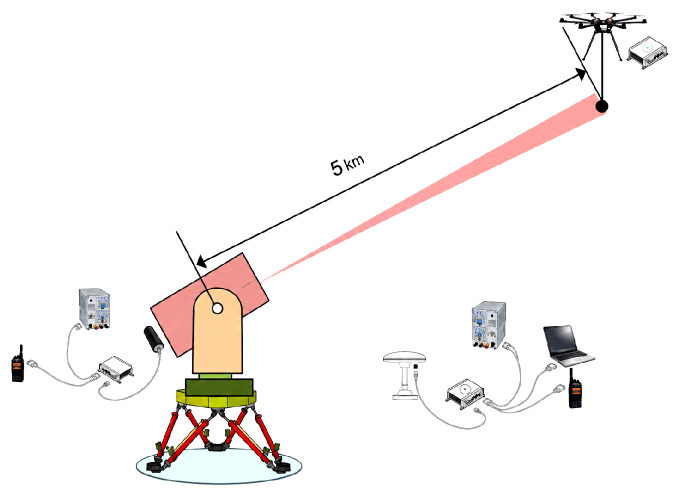
Schematic diagram of the laser ranging demonstration test verification platform.

**Figure 14:**
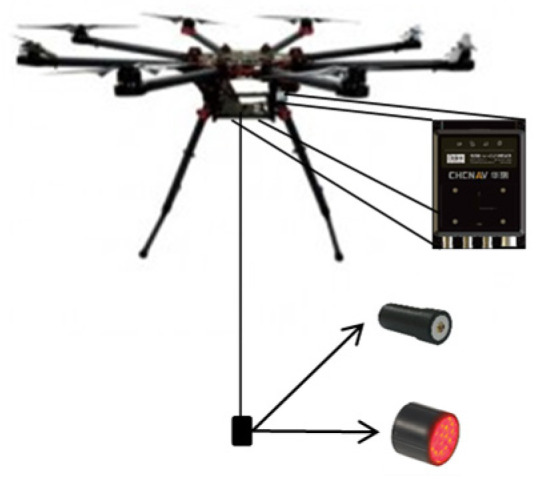
Unmanned aerial vehicle experimental device.

**Figure 15:**
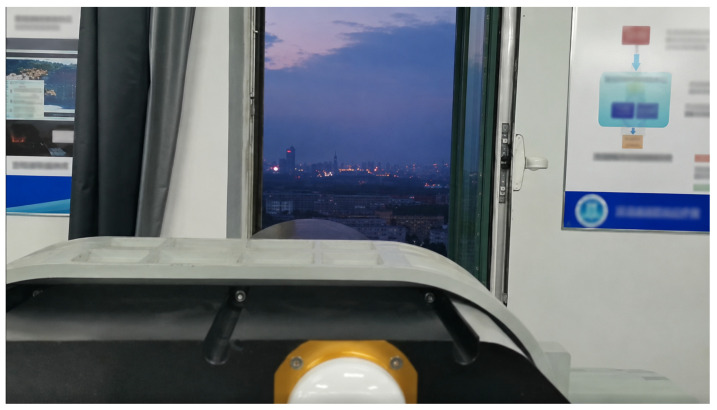
Drone trajectory obtained by the positioning system.

**Figure 16:**
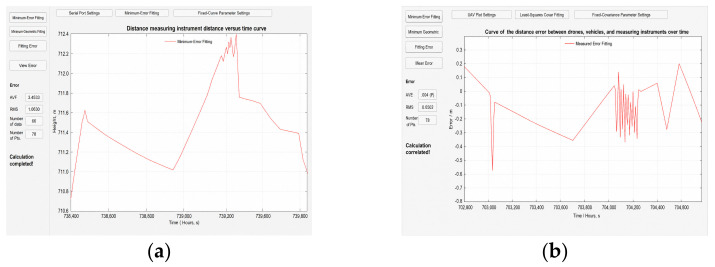
Ranging data acquisition and software processing: (**a**) partial distance data acquired by the ranging unit; (**b**) processed distance data obtained by the software.

In the ground experiment, an unmanned aerial vehicle (UAV) carrying a cooperative target was used for dynamic ranging (Figure 13). The UAV experimental device is shown in Figure 14. The drone trajectory recorded by the positioning system is shown in Figure 15, and the ranging data acquisition and software processing results are shown in Figure 16. The flight parameters of the UAV are: altitude H = 50–500 m, horizontal velocity v = 1~10 m/s, and closest slant range L ≈ 500 m. Under these conditions, the maximum measured angular velocity of the target relative to the system is:

(22) $$\omega_{\exp}\approx \frac{v}{L}=\frac{10\;m/s}{500\;m}=20\;\mathrm{mrad}/s$$

The angular velocity applied in the ground experiment, $\omega_{exp}$ = 20 mrad/s, is significantly higher than the system design index ω = 7.85 mrad/s. Despite the higher experimental angular velocity, the system successfully completed continuous tracking and ranging.

### 4.4. Laser Communication Results

#### 4.4.1. Experimental Setup

**Figure 17:**
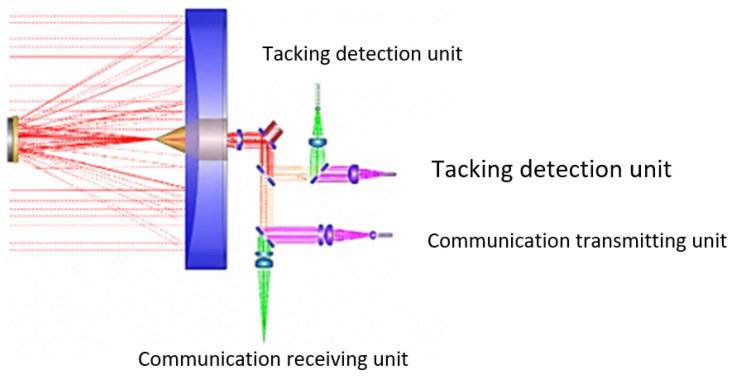
Optical path diagram of laser communication terminal.

The spatial optical path used in the experiment can be constructed by a pair of communication optical transceivers, whose optical principle is illustrated in Figure 17. The optical antenna of the transceiver is capable of compressing the beam divergence angle to less than 200 μrad, ensuring long-distance beam transmission. In addition, the transceiver integrates a coarse–fine composite-axis acquisition and tracking system with a tracking accuracy better than 25 μrad. This system features the function of acquisition and alignment between the two transceivers, which can ensure the alignment of the communication optical axes over an atmospheric channel with a distance of more than 1 km, thus facilitating the successful completion of the experimental work.

**Figure 18:**
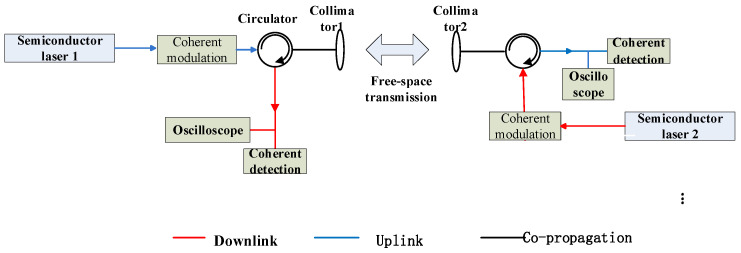
Schematic diagram of bidirectional high-order modulation spatial laser transmission.

The principle of bidirectional transmission of high-order modulated optical signals is shown in Figure 18. A single-longitudinal-mode laser operating in the 1.55 μm communication band is selected as the optical carrier. Laser LD1 modulates the high-order coherent coding for downlink transmission, while laser LD2 modulates the high-order coherent coding for uplink transmission. The bidirectional transmission is multiplexed and demultiplexed via a circulator, sharing the same optical system.

**Figure 19:**
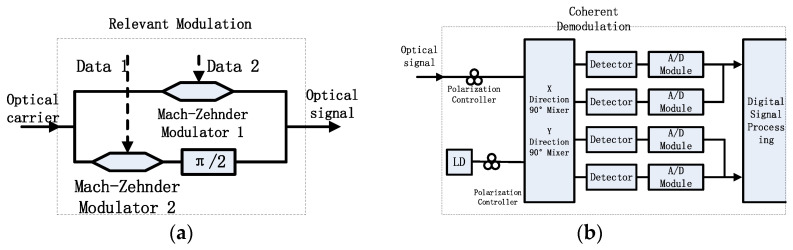
QPSK coherently coded signal: (**a**) modulation diagram; (**b**) demodulation diagram.

To realize high-speed spatial laser communication with high-order modulation, high-order coherent encoded signals such as QPSK and M-QAM with a data rate of over 20 Gbps are adopted [30]. A QPSK signal carries four phase states, namely 0, π/2, π, and 3π/2, which is generated by parallel modulation using an IQ modulator (i.e., three nested Mach–Zehnder modulators), as illustrated in Figure 19a. Each arm of the main modulator incorporates a Mach–Zehnder modulator biased at the null point, which can generate chirp-free, equal-intensity 0-π phase-shifted signals driven by OOK signals. By biasing the phase of one channel by π/2, a QPSK signal is obtained at the output of the main modulator, whose data rate is twice that of the input OOK signal. Therefore, a 10 Gbps OOK signal can be used to generate a 20 Gbps QPSK signal.

M-QAM signals can be generated by driving the IQ modulator with multi-level electrical signals produced by an arbitrary waveform generator, where a larger number of electrical levels corresponds to a higher M value. Demodulation of QPSK and M-QAM signals requires coherent detection, as shown in Figure 19b. The signal light and the external local oscillator light undergo phase-diversity coherent mixing via a 90° hybrid coupler, and the mixed signals are then received by four balanced detectors. A real-time oscilloscope is used to sample the data and convert the analog signals into digital signals. Subsequent offline digital signal processing (DSP) on the collected data enables the demodulation of QPSK and other signals [29], yielding performance parameters such as constellation diagrams and bit error rates (BERs). The signal eye diagram can be directly observed on the received optical signal using an eye diagram analyzer [31,32].

**Figure 20:**
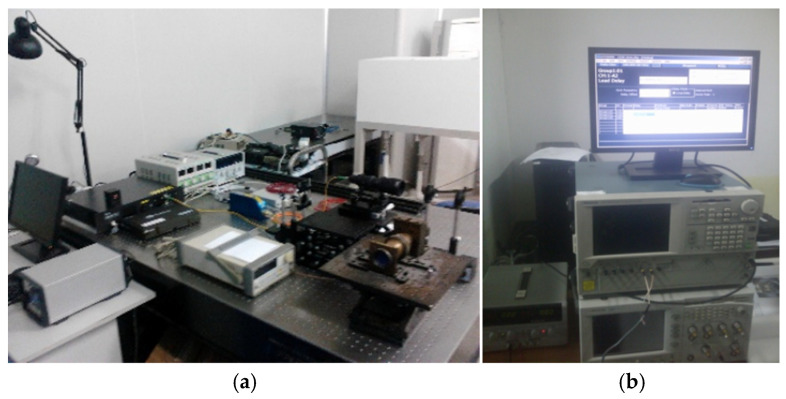
Experimental link system. (**a**) Laser communication transmitter setup; (**b**) laser communication receiver assembly.

The laser source in Figure 20a is transmitted through an optical lens after modulation and amplification and then received by a Cassegrain telescope system for communication laser reception, as shown in Figure 20b, establishing a laboratory-simulated laser communication link with a rate of 20 Gbps and a bit error rate (BER) of 10^−7^.

#### 4.4.2. Experimental Results

It should be noted that the link budget analysis in Section 3.3 and the ground communication experiment in this section correspond to different validation levels. The APD-based link budget is used for system-level feasibility estimation, whereas the 1 km coherent QPSK experiment is used to verify the high-speed modulation, free-space transmission, coherent reception, and offline demodulation capability of the communication module. Therefore, the 1 km experiment is not treated as a direct one-to-one verification of the 10 km APD link budget, but as a functional validation of the high-speed communication subsystem.

**Figure 21:**
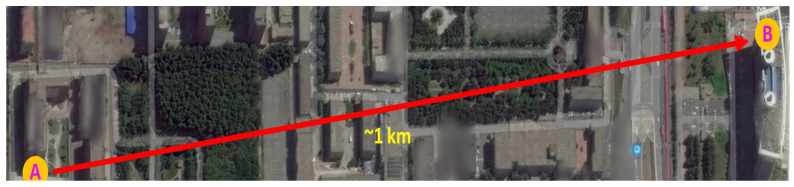
High-speed coherent laser communication test site map at the Changchun University of Science and Technology campus.

Experimental research on high-speed coherent laser communication was carried out between buildings on the Changchun University of Science and Technology campus. As shown in Figure 21, the laser communication terminal was placed in the Second Teaching Building (A) on the East Campus and the Science and Technology Building (B) on the South Campus of Changchun University of Science and Technology.

The experiment employed 20 Gbps high-speed QPSK optical modulation. The transmitter was located at point A, and the transmitted signal was measured using a real-time digital oscilloscope (Tektronix OM4006D, Inc., Beaverton, OR, USA) with a bandwidth of 23 GHz. The receiver at point B had an average received optical power of 9 dBm, and the transmission distance between the two points was 1 km. Under normal conditions, the transmission attenuation was 21 dB, mainly caused by free space and fiber optic device coupling, as well as atmospheric transmission attenuation. A narrowband filter with a bandwidth of 0.8 nm was used at the receiver to remove noise. After coupling between multi-mode and single-mode fibers, the detector received a power of approximately −21 dBm. The coherent detection system used a 100 kHz linewidth tunable distributed feedback semiconductor laser as the local oscillator. A 90-degree optical mixer and two balanced detectors received the mixed light. The electrical signal from photodetection was then amplified by a low-noise preamplifier and input to a four-channel broadband real-time digital oscilloscope (Tektronix DPO72004C Inc., Beaverton, OR, USA) with a bandwidth of 20 GHz and a sampling rate of 100 GS/s. Offline demodulation algorithms were used to analyze the signal.

**Figure 22:**
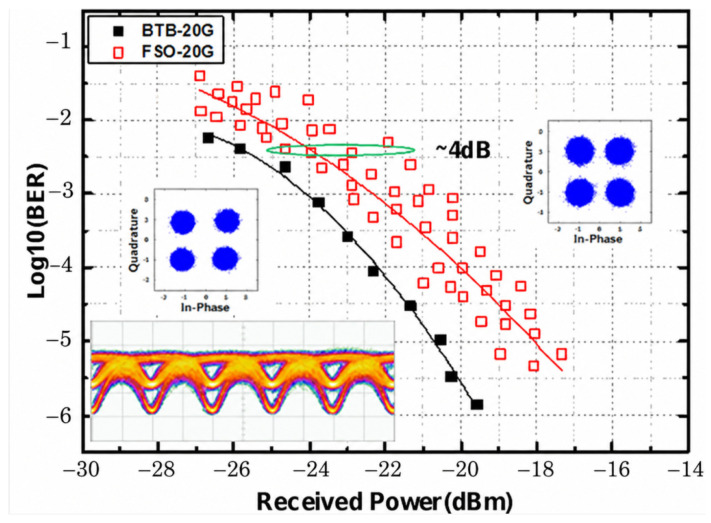
BER performance of 20 Gbit/s QPSK coherent signals under back-to-back (BTB) and free-space optical (FSO) transmission, with representative constellation diagrams and eye diagrams.

As shown in Figure 22, the constellation diagram and eye diagram of a 20 Gbps QPSK modulated signal were measured back-to-back and synchronously with the received signal. The bit error rate characteristics were obtained using an offline algorithm. Calculations based on the back-to-back constellation diagram showed a mean square error vector amplitude of 23.2%, which increased to 25.8% after spatial optical transmission with low attenuation, indicating a 2.6% increase in phase ripple. The bit error rate increased with signal attenuation. During the experiment, the bit error rate remained essentially linear when the received power of the QPSK signal fluctuated by approximately 4 dB.

This communication experiment verified the high-speed modulation, free-space transmission, coherent reception, and offline demodulation capability of the communication module under the tested 1 km ground link. The results show that 20 Gbps QPSK signal transmission was achieved with a bit error rate on the order of $10^{-7}$ under the tested conditions. Together with the link budget analysis, the experiment suggests the potential of the proposed optical configuration for longer-distance applications, while further receiver-specific link budget verification and long-range dynamic communication tests are still required.

**Figure 23:**
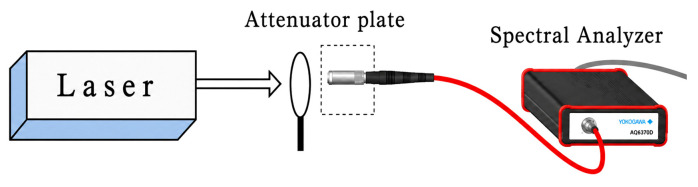
Experimental setup of the laser communication test.

**Figure 24:**
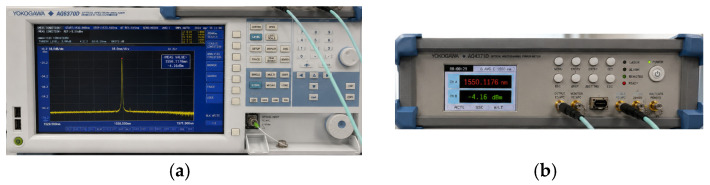
Experimental results of the laser communication module: (**a**) communication wavelength measurement results; (**b**) bit error rate (BER) test results.

The test setup is shown in Figure 23, and the corresponding communication wavelength and bit error rate (BER) measurement results are shown in Figure 24.

The ground experiments in this work were designed to verify the functional feasibility of the proposed integrated imaging, ranging, and communication architecture rather than to fully reproduce all space target observation conditions. The scaled optical equivalence analysis provides a basis for comparing angular size, target acquisition, optical throughput, and tracking feasibility under a controllable near-ground atmospheric path. However, the near-ground atmosphere, turbulence distribution, background illumination, target reflectance, and relative motion characteristics are not completely identical to those in spaceborne or space-to-target scenarios. Therefore, the experimental results should be interpreted as system-level functional validation and feasibility evidence rather than exhaustive qualification of the system for all space conditions.

For polarization imaging, the reported contrast values were obtained from representative field images using consistent target and background regions. Since the experiments were conducted under natural haze and illumination conditions, the results demonstrate the contrast enhancement potential of the polarization channel, while more systematic measurements under controlled visibility, illumination, and repeated trial conditions are still required. For dynamic ranging, the UAV-based experiment verified acquisition, tracking, and ranging of a moving cooperative target, but a complete statistical error distribution requires larger sample numbers and independent reference measurements. For laser communication, the link budget analysis and the 1 km coherent QPSK experiment correspond to different validation levels. Future work will further integrate receiver-specific link budget verification, long-distance dynamic communication tests, and more rigorous field trials under controlled atmospheric conditions.

## 5. Conclusions

This paper conducts an in-depth study on the integrated technology of laser imaging, ranging, and communication, proposes a system scheme based on modular design, and verifies its feasibility and effectiveness through a series of experiments. The system consists of a laser ranging and communication beacon subsystem, an information imaging and communication transmission subsystem, a laser detection and beacon, a signal light source, and a target acquisition, alignment, and tracking subsystem, which can realize the efficient integration of laser communication, ranging, and imaging functions [23]. By adopting fiber phased array beam splitting–coupling technology, the system realizes the integration of multi-wavelength, high-power, and high-stability light sources, which improves the compactness and integration level of the system.

The visible light polarization imaging experiments demonstrate that polarization-derived information can provide complementary contrast for target observation under haze and low-contrast atmospheric conditions. Representative field results show that the target contrast increased from 5.4% in the intensity image to 58% in the polarization image for the 3 km haze experiment and from 11% and 5% to 51% and 49% for the 1.5 km summer and winter scenes, respectively. These results support the effectiveness of the polarization imaging channel for improving target–background separability under degraded visibility conditions.

The UAV-based dynamic ranging experiment verified that the integrated system can acquire, track, and range a moving cooperative target under the tested field conditions. The measured results are consistent with the designed meter-level ranging requirement. Since the field test was mainly designed for functional verification, a complete statistical characterization of dynamic ranging error will be carried out in future work using larger sample numbers and independent reference measurements.

Finally, the communication experiment verified the high-speed coherent transmission capability of the proposed communication module. In the 1 km ground free-space link, 20 Gbps QPSK signal transmission was achieved with a bit error rate on the order of 10^−7^. The link budget analysis suggests that the proposed optical configuration can provide a positive power margin for a 10 km-class link under the assumed receiver conditions. However, further long-distance experiments and receiver-specific link budget verification are still required before extending the conclusion to practical spaceborne links.

Overall, the main contribution of this work is the coordinated integration and experimental verification of visible light polarization imaging, pulsed laser ranging, and coherent laser communication within a unified optical terminal architecture. Rather than demonstrating isolated single-function performance, this study verifies the cooperative operation of the three functional channels through ground-based experiments, including kilometer-level polarization imaging, UAV-based dynamic ranging, and 20 Gbps coherent free-space communication. These results provide a design reference for compact multi-functional optoelectronic terminals under SWaP-constrained platform conditions, while more systematic space-equivalent validation remains necessary in future work.

## Figures and Tables

**Table 1:** Optical power budget calculation table for master optical terminal transmission—slave optical terminal reception.

System Parameter	Value	Note
Communication transmitting power	30 dBm	1 W laser adopted
Transmitting optical path loss	−1.55 dB	Transmitting optical efficiency 0.7
Atmospheric loss	−3 dB	Atmospheric transmittance 0.5
Space loss	−23.98 dB	10 km, transmitting angle 0.3 mrad, effective receiving aperture 200 mm
Receiving optical path loss	−1.55 dB	Receiving optical efficiency 0.7
Received power at APD	−0.08 dBm	Actual power arriving at APD detector
APD detection sensitivity	−20 dBm	APD limit sensitivity at BER = 10^−7^, 20 Gbps
Safety margin	19.92 dB	Margin above 3 dB reserved

**Table 2:** Equivalence assumptions and validity constraints of the scaled ground experiment.

Item	Existing Basis in This Work	Role in Equivalence Analysis	Validity Constraint
Scaling ratio	1:10 equivalent scaling	Used for principle verification	Not a full replication of the space environment
Target size and distance	5 cm target at 5 km	Angular size equivalent verification	Mainly valid for imaging and tracking principle verification
Atmospheric transmittance	MODTRAN result: transmittance > 90% within 5 km in clear weather	Supports ground-path feasibility	Limited to clear-weather or high-visibility conditions
Optical aperture	Space system aperture estimated as 1.2 m; scaled system used for ground verification	Supports aperture-scaling discussion	Does not fully represent all spaceborne aperture and payload constraints
Turbulence and scattering	Near-ground path includes atmospheric influence	Treated as environmental degradation	Not equivalent to all space-to-ground or spaceborne paths
Angular motion	UAV/swing table dynamic test	Used as tracking and ranging stress test	Not identical to orbital relative motion
Received energy	Evaluated through ranging/communication energy models	Supports feasibility analysis	Requires further validation for long-distance space links

**Table 3:** Modeled uncertainty budget of single-shot laser ranging.

Serial Number	Error Source	Error Types	Calibration Error Value (m)
1	Pulse width error	Random error	1.15~2.02
2	Light emission delay error	Random error + systematic error	0.02
3	Optical system error	Random error + systematic error	0.002
4	Detector delay error	Random error + systematic error	0.03
5	Timer error	Random error + systematic error	0.03
6	Calibration error	Random error	0.075
7	Platform motion error (optical travel time error)	Random error + systematic error	0.01
8	Platform rotation error	Random error + systematic error	0.001
Total error after compensation			1.16~2.02

## Data Availability

Data underlying the results presented in this paper are not publicly available at this time but may be obtained from the authors upon reasonable request.
